# Nothing in cancer makes sense except…

**DOI:** 10.1186/s12915-018-0493-8

**Published:** 2018-02-21

**Authors:** Mel Greaves

**Affiliations:** Centre for Evolution and Cancer, The Institute of Cancer Research, Brookes Lawley Building, 15 Cotswold Road, Sutton, Surrey SM2 5NG UK

## Abstract

Paraphrasing Dobzhansky’s famous dictum, I discuss how interrogating cancer through the lens of evolution has transformed our understanding of its development, causality and treatment resistance. The emerging picture of cancer captures its extensive diversity and therapeutic resilience, highlighting the need for more innovative approaches to control.

## Evolutionary biology and medicine

Dobzhansky’s insight applies not just to biology but to much in medicine. For example, our vulnerability to many chronic diseases in modern societies probably owes much to a mismatch between contemporary lifestyles and historical, evolutionary adaptations [1, 2]. Another potent example is with the development of drug resistance in microbes and parasites which is contingent upon clonal, evolutionary selection [3]. Similarly, the emergence of new or more virulent microbial pathogens reflects the outcome of evolutionary arms races between the immune system’s pathogen recognition repertoire and the high mutability of viruses, parasites and bacteria [4]. It’s a travesty that it is still possible to obtain a medical degree whilst in denial, or lacking understanding, of the essential tenets of evolutionary biology [5]. But, it is also likely that some evolutionary biologists are unaware of the medical implications of their field.

**Fig. 1.**
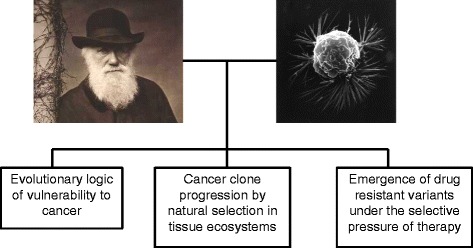
Nothing in cancer makes sense except in the light of evolution. Images: Charles Darwin (Cambridge University Library), breast cancer cell (National Cancer Institute [83])

Cancer provides a paradigm for the applicability of evolutionary principles to a medical problem [6]. An appreciation that cancer clones develop, or evolve, in the context of a complex tissue ecosystem has transformed our understanding of cancer biology and highlighted the need for more innovative approaches to therapy that can thwart evolutionary resilience [7–9]. An evolutionary logic pervades all major areas of cancer sciences including causation, cancer clone development and resistance to therapies [10] (Fig. 1).

## Cancer clone evolution

**Fig. 2.**
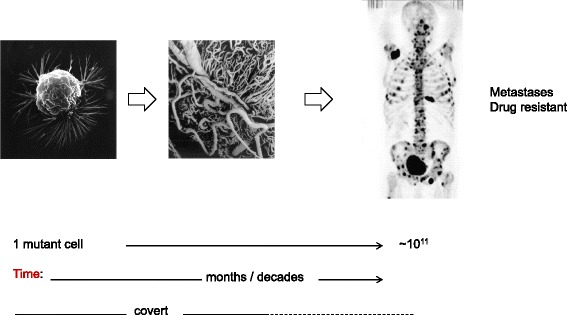
Natural history of cancer. *Left*: Breast cancer cell (National Cancer Institute [83]) *Middle*: Stereoscan image showing neovascularisation around an *in situ* carcinoma (angiogenesis). Photo provided by Professor M A Konerding. *Right*: PET scan showing cancer disseminated throughout the body (*dark patches*). Image originally published in *JNM* [84] and reproduced with permission: Even-Sapir E, Metser U, Mishani E, Gennady Lievshitz G, Lerman H, Leibovitch I. The detection of bone metastases in patients with high-risk prostate cancer: 99mTc-MDP planar bone scintigraphy, single- and multi-field-of-view SPECT, 18F–fluoride PET, and 18F–fluoride PET/CT. *J Nucl Med.* 2006;47:287–97. © by the Society of Nuclear Medicine and Molecular Imaging, Inc. Most of this evolutionary process is clinically silent or covert

The natural history of cancer is illustrated in a very simplified fashion in Fig. 2. The evolutionary trajectory of a cancer clone, starting from a single mutant cell and progressing to a malignant and metastatic clone of ~ 10^11^ cells, can have very variable dynamics, with time frames ranging from a few months (some aggressive paediatric tumours) to one or several decades (many adult epithelial carcinomas). The tempo of cell population dynamics can be steady or proceed in jumps—punctuated equilibrium [11]. The majority of initiated tumour clones never evolve to fully fledged malignant clones [12, 13] but for those that do, the end game is dissemination in the body, or metastasis, a territorial hijack with onboard therapeutic resistance. It’s an evolutionary process, not just in terms of change over time but in the true Darwinian sense of random genetic variation and natural selection of the best-adapted or fittest variants. Cancer clone progression is equivalent to fast track evolution of an asexual species of unicellular organisms. But it’s fuelled by the recombinatorial genetic diversity normally acquired via sexual reproduction.

These ideas first emerged in the 1970s [14]. The evidence then was based on observations of serial changes, over time, in gross chromosomal structural alterations in cells. The current perspective is more detailed and contextual [7] with cancer cells subject to whole genome sequencing [15]. Single cell genetic scrutiny [16–20] or analysis of small micro-dissected regions of tumours [21–23] identifies sub-clonal architectures from which phylogenic relationships can be inferred.

**Fig. 3.**
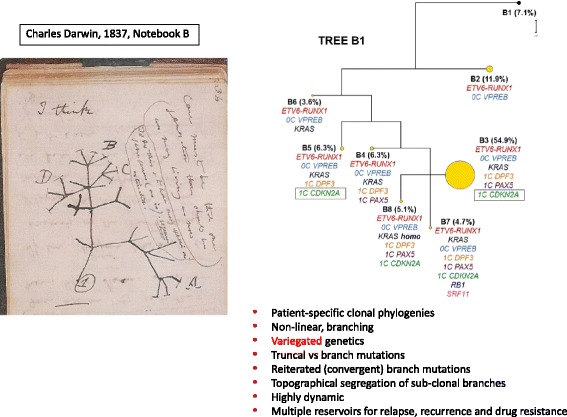
Critical features of cancer clone phylogenetics. *Left*: Charles Darwin’s iconic ‘I think’ drawing of a phylogenetic tree from his 1837 Notebook (B) [85]. *Right*: Example of subclonal phylogeny based on single cell genetics (in leukemia), taken from author’s own research in [17]. Seven subclones shown (*B2–8*), each with mutations listed. B1 (7.1%) are normal cells. *CDKN2A* in *green* in *box*: reiterated mutation of same gene is different branches or subclones. One subclone (*B3*) is numerically dominant (at 54.9%)

Clonal phylogenies for cancer cells can reveal early or founder genetic lesions (present in all cells) and time-ordered sequences of subsequent mutations. In most cases, sub-clonal architectures are branching rather than linear [15–18, 21–24], reminiscent of Charles Darwin’s iconic ‘I think’ drawing in which he imagined how different species might evolve from a common ancestor (Fig. 3). Side branches of individual cancers often have independent mutations in the same genes, reflecting parallel or convergent evolution and prevailing selective pressures on all sub-clones. This new, evolutionary portrait of cancer cell diversity and its variegated genetics [24] has considerable practical implications for patient prognosis, monitoring and treatment [8–10].

There are caveats to these analyses. Cancer cell phylogenetic constructs are often based on single time point snapshots and miss the dynamic shifts in sub-clonal population structure that occur at early pre-clinical time points, over time with progression of disease and in recurrence or relapse. The depth of genomic sequencing is still limited in most cases and, as a consequence, minor sub-clones are invisible and the extent of diversity under-estimated [25].

Although some of the sub-clonal architecture in cancer derives from neutral evolution or drift [26], particularly in early phases with low cell numbers [27], a prevailing view is that cancer cell populations undergo positive, or, occasionally, negative, selection via tissue ecosystem pressures [7, 28]. In this sense, the highly recurrent genetic changes in gene copy number or single nucleotide variants can be seen as adaptive, being selected, in a Darwinian sense, as a consequence of the fitness benefit they provide [10]. Fitness is expressed via so-called ‘hallmark’ phenotypic features of cancer cells [29], which include enhanced proliferation, resistance to signals for cell death or senescence, metabolic changes and epigenetic shifts favouring self-renewal of stem/progenitor cells at the expense of differentiation. All of which impact, directly or indirectly, on reproductive fitness. Some cancers exhibit massive genomic instability [15] but even this can be considered as an adaptive strategy, gambling on rare ‘winners’, as similarly employed by bacteria under potent metabolic stress [30].

## The cancer ecosystem

The cancer tissue ecosystem is itself complex and dynamic and is altered as a consequence of the invasion of cancerous cells. Understanding the network of regulatory interactions within the cancer ecosystem, involving stromal cells, the vasculature, and invasive inflammatory cells, is in its infancy. Nevertheless, there is accumulating evidence that features of the cancer microenvironment, including hypoxia and acidosis, diversity of inflammatory cell infiltrates, activated stromal cells and patterns of vascularisation, are major drivers of cancer clone progression, impacting on clinical outcome [24, 27, 31–35]. Ecosystem variables may also provide novel opportunities for therapy [36].

These considerations suggest that it might be possible to define an evolutionary and ecological index of individual cancers that is predictive of the likelihood of progression, metastasis and drug resistance and which could, in the future, guide critical patient management decisions [37].

Not all cancer cells have equivalent proliferative capacity. Many cancers, if not all, have sub-populations of cells with stem cell-like features or self-renewal capacity, i.e. they reproduce themselves at the expense of differentiation [38]. The frequency or proportion of these cells within a cancer cell population varies greatly, as do their other phenotypic features, which has led to some confusion on their relevance. However, most cancer sub-clones contain cells with extensive propagating or stem cell function [24, 39–41]. It is likely that self-renewing, or stem, cells are the major cellular substrate for the selective processes that underlie clonal architecture, progression of disease, metastasis, recurrence and drug resistance. As such, they provide both the evolutionary units of selection [42–44] in cancer and the ultimate targets for therapeutic control or cure [10, 38, 45, 46].

Technical advances in genomics have driven much of this paradigm shift in our understanding of cancer biology. On occasion, this has encouraged a rather gene-centric view of cancer, not altogether dissimilar to Richard Dawkins’ selfish gene perspective on evolution itself. Cancer has, for example, been defined as ‘a disease of the genome’ [15]. Mutated genomes lie at the heart of the emergence and malignant progression of cancer but we should not ignore the critical, contextual role of the ecosystem habitats in which this adaptive process occurs.

Mapping cancer clone complexity and evolution over time is demanding with solid tumours, which, unlike the blood borne leukaemias, often have topographically segregated sub-clones [21–23]. This means that biopsy-based samples can be highly biased [8]. A solution to this challenge is, however, provided by serial screening of cancer-derived DNA fragments in plasma. This allows presumed unbiased, sensitive, serial monitoring of cancer clone evolution, virtually in real time. Applications of this technology are already impacting on patient management, for example by detecting the early, pre-clinical re-emergence of disease or drug-resistant sub-clones [47–49].

## Evolutionary origins of vulnerability to cancer

There is less appreciation of the relevance of an evolutionary perspective to causation in cancer. Cancer epidemiologists, understandably, focus on proximate mechanisms that hold the prospect of intervention—for example with cigarette smoking, UVB exposure or viruses. It is now well recognised, from genome-wide association studies (GWAS), that a very large number (~ 100 s) of inherited gene variants impact on cancer risk. Individual variants are mostly in non-coding regulatory regions and, individually, contribute to only a very modest increase in risk, conferring odds ratios of ~ 1.01–1.1 [50].

What is missing from this genetic epidemiology description is an appreciation of vulnerability. Why is it that animals in effectively all phyla can, and do, develop cancer [51], including Cnidaria (e.g. Hydra) at the base of the animal phylogenetic tree [52]? Why is it that cancer risk for ageing humans is now off the scale at close to a 50% lifetime risk?

The ubiquity of cancer can plausibly be ascribed to the intrinsic ‘design’ fallibilities of replicating organisms [53], including the essential mutability of DNA. Many cancer relevant genes recognised as tumour suppressors or oncogenes appeared at that time that multi-cellularity emerged around 700 million years ago [54]. The multi-cellular contract requires compliance of cell behaviour and proliferative restraint. But as many cells, and especially stem cells, retain extensive proliferative or regenerative capacity, opportunities exist for mutant cheaters. And, as in other social groupings [55, 56], cheaters will occasionally succeed in taking these opportunities. Stem cells, in particular, are an evolutionary liability with respect to mutability, positive selection and cancer [57]. Moreover, they function in an environment, endogenous or exogenous, replete with genotoxic challenges that can damage DNA as with, for example, UVB, natural plant toxins or oxidative metabolism.

But we still require a plausible explanation of why humans, at least in modern, developed societies, have such a high lifetime risk of a cancer diagnosis. Some ascribe this to a consequence of ageing itself. As common, historical causes of death—famine, predation, infection, cardio-vascular disease—have come under control, cancer could then be the default health outcome in ageing individuals who are post-reproductive and may have reduced capacity for DNA repair and immune surveillance. Could this just be then the legacy of intrinsic cancer risk leaking through unrestrained? If so, how come ageing and large elephants and whales have rather little cancer (Peto’s paradox) [58, 59]? How come the incidence rates of particular cancers vary substantially (2–400×) between different places and over time, and as seen in migrant groups [60]? The time/place patterns of cancer incidence only make sense if lifestyle factors are a significant component of risk.

**Fig. 4.**
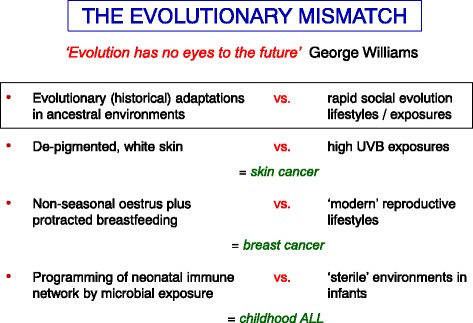
Evolutionary origins of vulnerability to cancer. See [63] for detailed discussion of this argument

One explanation for at least some of the very high risk of cancer in humans that this author favours is that it reflects the impact of chronic exposure to an evolutionary mismatch [6, 53]: a mismatch between our rapid social evolution and ‘modern’ lifestyles versus historical, evolutionary adaptations (Fig. 4). An example of this would be with the risk of skin cancers in white, Caucasian individuals, which is orders of magnitude greater than the risk of individuals with black or pigmented skin. Historically, variable levels of skin pigmentation, via melanin content, have been selected by environmental pressures. Depigmented, pale or white skin was an adaptation to cloudy northern climes with low UVB levels in Europe, improving survival prospects and reproductive fitness probably via enhanced vitamin D levels and, possibly, diminution of the impact of frostbite [61]. Modern lifestyles and foreign exile or travel have disrupted this adaptive context. This is rapid social evolution that outpaces any prospect of genetic, evolutionary adaptations. Similar arguments apply to modern reproductive lifestyles (compounded with diet)—early menarche, delayed first pregnancy and diminished breastfeeding that escalate risk of breast cancer via a mismatch with evolutionary adaptations of non-seasonal oestrus and protracted breastfeeding [62, 63]. Reduced microbial exposures in infancy may underlie the increased risk of childhood acute lymphoblastic leukaemia in affluent, developed societies as they deprive the immune system of the ‘educational’ microbial exposure required for its network settings [63].

## Ways of escape

When cancer is lethal, this is usually because of two reasons. Firstly, the cancer clones disseminate or metastasize to ecotopic tissues, compromising essential functions. And, secondly, at this advanced stage cancer cells are almost invariably robust and resistant to therapy. Thwarting the evolutionary resilience of cancer can then be seen as the major therapeutic challenge [10, 64–66].

The emergence of drug resistance makes sense, and only makes sense, in an evolutionary context. Several distinct routes to therapeutic escape are employed and each has an evolutionary rationale (Table 1). The ‘classic’ route to drug (or immunotherapy) resistance in cancer is, essentially, via the same mechanisms as seen with drug resistance in bacteria, malaria or HIV [3, 67, 68]; namely the positive selection (by therapy) of pre-existing variants that can evade the drug, or immune predation, via the serendipitous possession of mutations in the target pathway. We don’t yet have a Luria-Delbrück fluctuation test for this. Nevertheless, with highly targeted therapy, escapee cells spawning recurrence of disease have mutations in drug binding sites and can be backtracked, in some instances, to tissue prior to drug exposure [69–71]. Given enough cells and a reasonable mutation rate, this route to escape is inevitable.

**Table 1:** Routes to escape from targeted therapy in cancer

• Genetic variation	i. ‘Target’ segregated in sub-clones, not truncal
	ii. ‘Target’ mutated and impervious to drugs
• Epigenetic plasticity	i. Inhibited target bypassed by signal redundancy in network
	ii. Quiescent/dormant stem cells intrinsically resistant

If the therapeutic target, e.g. a mutant kinase, is in a side branch of the cancer clone’s evolutionary tree rather than truncal, then targeted therapy with a tyrosine kinase inhibitor (TKI) cannot be curative [72]. It’s then equivalent to pollarding trees or pruning plants—the plant, or cancer, shrinks in size and then rebounds with a flourish. Therapies targeted at critical signalling molecules such as kinases are also readily bypassed by signal redundancy [73, 74], a longstanding, networked feature of eukaryotic cells [75].

As might have been anticipated from an evolutionary perspective, cancer stem cells can evade therapeutic predation via other routes. Normal stem cells spend most of their time reversibly quiescent and out of proliferative cell cycle, which renders them less liable to mutation and damage [76]. They are also well equipped with efflux pumps for noxious, drug-like natural chemicals. Stem cells are vital to life—and limited in number. It is unsurprising that they will have acquired multiple protective mechanisms; mechanisms that can be readily co-opted by cancer stem cells under therapeutic assault [77]. It is now clear that quiescent or dormant cancer stem cells are intrinsically resistant to drugs and radiotherapy [38]. They can ‘hunker’ down, using a ploy long adopted by bacteria and other, eukaryotic, unicellular species [78]. Dormant cancer stem cells can re-enter a proliferative cycle and regenerate a malignant clone after two decades or more of ‘sleep’ [79].

Collectively, these escape routes equip cancer cells, especially in advanced disease with high turnover burdens and high mutation rates (or genetic instability), with great resilience (Table 1). Like a tardigrade [80], they can survive almost any insult.

The one real success in targeted therapy, small molecule tyrosine kinase inhibitors (TKIs) in chronic myeloid leukaemia (CML) [81], is contingent upon the target kinase (ABL1) being the founder lesion in every cell *and* the clones having minimal genetic diversity. Resistant mutants with altered ABL1 kinase do occur (prior to treatment) but many patients achieve sustained remissions or re-enter remission following a switch to an alternative TKI [71]. And when patients are effectively ‘cured’, the mutant leukaemia stem cells may persist for a protracted period of time, but in a dormant state. CML stem cells that are quiescent, or dormant, appear to have no dependence on the mutated ABL1 kinase. The CML clone isn’t then eradicated but kept under control, its capacity for evolutionary progression blocked.

## Outlook: strategies for control

The extent of cellular and genetic diversity in cancer, both between and within individual patients, is daunting but has a logic in terms of normal tissue developmental biology and evolutionary processes of drift and selection [10, 28]. We now see that the major challenge in cancer control is how to thwart the evolutionary resilience of the disease, especially when it is detected relatively late in its trajectory, as with pancreatic, CNS, lung and ovarian cancers. In principle, several strategies are available to us, all endorsed by the National Cancer Institute in the USA and Cancer Research UK. First, despite the contentious argument that many cancers arise via spontaneous mutation [82], the majority are potentially preventable. For example by prudent avoidance (e.g. cigarettes, solar UVB), modified behaviour (e.g. diet/exercise balance) or prophylaxis (e.g. virally induced cancers such as HPV/cervical cancer). Secondly most cancers are curable by surgery or radiotherapy if detected early when localised.

The main challenge of contemporary cancer therapeutics is with advanced or metastatic disease. The third essential strategy is to design drug combinations, including immunotherapy, and schedules that can thwart the emergence of resistance in established disease [64] by either eliminating all cancer stem cells, steering them into more benign fitness peaks or applying an evolutionary break [65]. ‘Taming’ may be a more achievable objective than elimination for metastatic disease [64, 65]. Having a better understanding of the cancer ecosystem and its selective pressures might facilitate novel approaches to control that do not solicit emergence of resistant sub-clones.

**Fig. 5.**
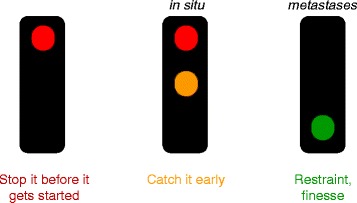
Traffic control of cancer cell evolution. *Red*: Stop it before it gets started: prophylactic intervention (e.g. HPV vaccines) or prudent avoidance (e.g. cigarette tar, UBV). *Red + amber*: Catch it early: by surveillance of populations or at risk individuals and early intervention by surgery or radiotherapy. *Green*: The cancer has already escaped. Employ therapeutic strategies to finesse or restrain continued growth and drug resistance. *In situ* refers to tumours confined to primary tissue site. *Mets* metastatic lesions – cancers disseminated to ectopic tissue sites, e.g. breast to brain, prostate to bone. Adapted from author’s reference [10]

There is not, and will not be, a magic bullet or penicillin equivalent for cancer. We need to intervene at all three time points in cancer’s evolutionary trajectory (Fig. 5) if we are to erode further its impact on society.
